# Dental careers: findings of a national dental workforce survey

**DOI:** 10.1038/s41415-024-8234-6

**Published:** 2025-02-28

**Authors:** Megan Clark, Ailsa McGregor, Aina Najwa Mohd Khairuddin, Malcolm Smith, Jennifer E. Gallagher

**Affiliations:** 41415334201001 Oral Surgery Specialist, Oral Surgery Department, Newcastle Dental Hospital, Richardson Road, Newcastle, NE2 4AZ, UK; 41415334201002 Associate Dentist, London, UK; 41415334201003 Postgraduate Researcher, Dental Public Health, Centre for Host Microbiome Interactions, Faculty of Dentistry, Oral and Craniofacial Sciences, King´s College London, Bessemer Rd, London, SE5 95S, UK; Lecturer, Department of Community Oral Health and Clinical Prevention, Faculty of Dentistry, Universiti Malaya, Kuala Lumpur, Malaysia; 41415334201004 Former Postgraduate Dental Dean, Former Chair of Dental Education Reform Programme and Directorate of Multi-Professional Dental Education, NHSE Education North East England and North Cumbria, UK; 41415334201005 Ambassador International, Engagement and Service, King´s College London, UK; Newland-Pedley Professor of Oral Health Strategy and Honorary Consultant in Dental Public Health, Dental Public Health, Centre for Host Microbiome Interactions, Faculty of Dentistry, Oral and Craniofacial Sciences, King´s College London, Bessemer Rd, London, SE5 9RS, UK

## Abstract

**Aim** To explore dentists' experiences of their professional careers and job satisfaction within the United Kingdom (UK) workforce.

**Methods** A cross-sectional survey of the national dentist workforce was conducted using an online questionnaire, informed by previous research. The anonymous online survey was conducted between February and May 2021, with ethical approval, via national gatekeeper institutions, and promoted through social media. Statistical analysis of the data was performed in SPSS.

**Results** Of the 1,240 respondents, 875 had completed 96% of the questionnaire, including providing demographic details, and were included for analysis. Almost half (46%) reported their career was ‘not as envisaged'. A majority (58%) of dentists reported that their career plans had changed and 40.2% reported planning on changing careers. Significant associations were found between an individual's career plan trajectories (‘as envisaged', ‘changed plan' and ‘planning on changing') and sex, ethnicity, job satisfaction, primary role settings, country qualification was obtained and duration of working experience. Men were significantly more likely to report their career was as envisaged. Job satisfaction was higher for those whose careers were as envisaged and had no plans for future changes.

**Conclusions** Careers were not necessarily as envisaged, with over half of the dentists surveyed changing their career plans over their working life. There was greater satisfaction among those whose careers were envisaged and had experienced career progression..

**Supplementary Information:**

Zusatzmaterial online: Zu diesem Beitrag sind unter 10.1038/s41415-024-8234-6 für autorisierte Leser zusätzliche Dateien abrufbar.

## Key points

- Many dentists' careers do not follow the path they originally intended, with over half of participants reporting changes in their career plans. This shows that career development within dentistry can be fluid.
- Dentists whose careers followed their initial plans experienced higher career satisfaction. This highlights the importance of realistic career expectations for long-term job fulfilment.
- Many factors can influence career satisfaction and trajectory, such as sex, ethnicity and job roles; however, there is a need to facilitate careers for those in remote/rural areas and for women.
- Adaptability and openness to career changes are crucial to career satisfaction and growth as many dentists have either changed their career plans or plan to in the future.

**Supplementary Information:**

Zusatzmaterial online: Zu diesem Beitrag sind unter 10.1038/s41415-024-8234-6 für autorisierte Leser zusätzliche Dateien abrufbar.

## Background

The United Kingdom (UK) has approximately 45,000 registered dentists,^1^ over half of whom are female.^2^ Dentists work across the four nations of the UK, mostly in primary care settings. About 10% of the profession have listed professional titles across 13 dental specialties.^1^ There are increasingly other options available to facilitate diversification, together with education, research, specialties or special interests, leadership and management,^3^ including expanding into the medical specialty of oral and maxillofacial surgery.^4^ Dental careers require regular consideration given the influence of national health and educational policies, patient and public needs and professional interests; however, research in this field is limited.

### Health workforce policies

All four nations of the UK have National Health Service (NHS) workforce strategies or reviews, which can help enable the effective management of the dental workforce to deliver the right care, at the right time, in the right place.^5^^,^^6^^,^^7^^,^^8^ In England, Advancing dental care (ADC),^9^^,^^10^^,^^11^ and Dental training reform,^12^ led by NHS England Education (formerly Health Education England [HEE]) have highlighted the importance of facilitating professional career development. In parallel, Health Futures work has explored how the workforce may be harnessed to address the oral health needs of our changing population.^13^

A similar theme of workforce retention and developing education and training opportunities exists across workforce reports for the other UK nations, with Scotland's latest report highlighting that the supply of dentists is likely to fall short of population demand, with uncertainty on the inflow of dentists from other non-UK sources, which may impact on the number of dentists in Scotland.^14^ Wales is also investigating enhanced recruitment offers to incentivise dentists to work in rural locations, with enhanced training opportunities and support.^15^ Northern Ireland's workforce report also discusses the workforce challenges, with increasing complexity and demand, resulting in increased pressures on the available workforce and resources for both dentists and other dental care professionals.^7^

Workforce planning is key to ensure we shape the right workforce to meet future population need, considering the NHS Five year forward view,^16^ the NHS Long term plan,^17^ NHS Workforce plan,^5^ and the English ADC review, emphasising the importance of looking at careers differently to help improve training and retain our dental workforce.^11^

### Professional careers

Much of the work on professional careers has emerged from the study of men in higher-income countries.^18^ In relation to dentistry, research by Gallagher *et al*.^19^^,^^20^^,^^21^^,^^22^^,^^23^ in the noughties investigated why final-year undergraduates and foundation dentists in England and Wales selected dentistry as a professional career, how they perceive their vision has changed during dental education, and their views on their future professional career plans and perceived influences. Longer-term professional expectations of foundation dentists were closely linked with their personal lives and supported a vision of a favourable work-life balance and ‘a professionally contained career in healthcare'.^19^

Consideration of the health and wellbeing of the dental workforce is also paramount when reviewing staff retention, with studies showing that health and wellbeing have a positive impact on patient care with improved performance from staff, and less sickness, staff turnover and absence rates.^24^ A growing body of evidence highlights the multiple determinants of dentists' health and wellbeing.^25^^,^^26^^,^^27^ The COVID-19 pandemic has appeared to heighten this challenge for health professionals.^28^^,^^29^

This is exacerbating pressures on the dental system,^30^ with significant workforce implications.^31^ A deeper understanding of the factors influencing the career progression of dentists and other members of the dental team is needed to optimise a sustainable future workforce.^32^^,^^33^

As our population ages, oral health changes and developments in science become available, the dental workforce needs to be able to adapt accordingly.^34^ This involves ensuring dental professionals have the skills, behaviours and support to provide high-quality, patient-centred care.^35^ Further education and training play an important role in professional career development and workforce retention. The NHS Dental Education Reform Programme,^12^ therefore, aims to engage with the workforce and key stakeholders to implement the recommendations of the ADC review,^9^^,^^11^ which include skills development, widening access and participation and flexible working.

In summary, considering the challenges to the health and wellbeing of the workforce, with the need to retain our workforce for the future, it is important to understand current professional career trajectories and to what extent these career aspirations have been or are being met.

The aim of this study was, therefore, to explore dentists' experiences of their professional careers and job satisfaction within the current UK workforce, together with their perceived barriers and/or facilitators to career progression.

## Method study design

This cross-sectional study represents a collaboration between the former HEE North East (now NHS England Education North East) and King's College London, Faculty of Dentistry, Oral and Craniofacial Sciences. Ethical approval was gained from King's College London Research Ethics Committee (No. MRA-20/21-22056).

### Questionnaire instrument

The questionnaire was developed within Qualtrics online survey software https://qualtrics.com/. It was based on previous instruments developed by the team at King's.^20^^,^^22^^,^^36^^,^^37^ Questions were adapted for the current context, to take account of organisational changes, as well as to accommodate both prospective and retrospective questioning, informed by national workforce policies.

The questionnaire,comprising open and closed questions and skip logic, explored the following: qualifications and role; work location; current role; career pathway; education and training; job satisfaction (score 0-10); barriers and facilitators to further training or education; and demographic information. The instrument was tested for face validity with experts in the dental workforce and piloted in advance with a convenient sample of dentists, following which questions were added on part-time and full-time working patterns and preferences, and the importance of different factors on working patterns. The questionnaire was also adjusted after piloting to account for the COVID-19 pandemic, to emphasise the difference this may have made to job roles. Finally, the questionnaire was split into sub-sections to aid flow:

- Section 1 - qualification details and geographic location
- Section 2 - current (pre-COVID-related changes) professional career within dentistry
- Section 3 - education and training
- Section 4 - demographics.

### Study population

All registered dentists in the UK were invited to participate in the survey, except for foundation dentists, who would have only just graduated/joined the profession and were in mandatory training and thus may still have been forming their career aspirations. This was explicit in the study information sheet.

### Recruitment

Dentists were invited to complete the initial questionnaire from the research team via a range of gatekeepers: British Dental Association (BDA), General Dental Council (GDC), Business Services Authority (BSA) and local dental networks. Additionally, the survey was promoted via social networking sites.

Initial signposting to the survey via gatekeepers was followed-up by a series of reminder emails or signposting, in line with Dillman's protocol for online surveys.^38^ This involved four reminder emails, with the final communication announcing the imminent closure of the questionnaire.

### Consent

The survey link was signposted through emails, news bulletins or websites via the gatekeepers, as outlined above, between February and May 2021. After clicking on the survey link, participants were provided with details of the study, including an information sheet, consent form and contact details of the lead researcher for any questions or complaints (questionnaire available on request). Participants were informed via the first page of the survey that submission of the questionnaire implied their consent for the data to be used in publications and to inform policy. They were also informed that once their response had been provided, it was not possible to withdraw from the study due to its anonymous nature.

### Analysis

Submitted data were extracted from Qualtrics into Excel and IBM SPSS Statistics version 29.0.10 (171). Respondents with incomplete information on sex were excluded from the analyses. Quantitative data were analysed using SPSS. The characteristics of the respondents from different career plan outcomes were compared in relation to sex, location, career stage, position and job satisfaction using chi-squared and Kruskal-Wallis tests. All categorical variables were reported by frequency (%), whereas numerical variables such as job satisfaction were reported by median and interquartile range, as they were not normally distributed. Multinomial logistic regression was used to estimate the association between potential predictors and career plan outcomes. The adjusted associations were reported as odds ratios (ORs) and 95% confidence intervals (CIs). Results with p-values less than 0.05 were considered statistically significant. Analysis of free-text responses will be presented in a future publication.

## Results

### Survey respondents

Overall, we received 1,240 responses, of which 875 had completed the demographic section on sex and were used in the analysis. Most participants were female (55.5%); white (75.4%); general dental practitioners (81.3%); working in England (91.7%); UK graduates (88.1%); and in primary care settings (62.9%) (Table 1).

**Table 1:** Characteristics of study participants (n = 875)

**Characteristics**	**n (%)**
**Sex**	
Male	366 (41.8)
Female	486 (55.5)
Not disclosed	23 (2.6)
**Ethnicity**	
White	619 (75.4)
Multiple ethnicity	16 (2.0)
Asian/Asian British	158 (19.2)
Black/African/Caribbean/Black British	11 (1.3)
Others	17 (2.1)
**Country obtained qualification**	
Within UK	769 (88.1)
Outside UK	104 (11.9)
**Duration of primary job role**	
0-5 years	318 (36.4)
6-10 years	117 (13.4)
11-15 years	95 (10.9)
16-20 years	237 (27.1)
21+ years	107 (12.2)
**Primary role setting**	
Primary care	550 (62.9)
Secondary care	148 (16.9)
University	56 (6.4)
Community dental services	88 (10.1)
Armed forces/other	33 (3.8)
**Place of current work**	
Within UK only	850 (97.7)
Outside and within UK	8 (0.9)
Not currently in employment	12 (1.4)
**Experience working in other places**	
Within UK only	177 (20.9)
Outside and within UK	143 (16.9)
Nowhere else	527 (62.2)
**Current main role**	
General dental practitioner	447 (81.3)
Dentist with special interest/extended skills	68 (12.4)
Specialist	24 (4.4)
Other	11 (2.0)
**Further roles in other settings**	
No further roles	517 (62.1)
1 role	272 (32.7)
≥2 roles	43 (5.2)
**Employment**	
Full-time (5 days a week)	449 (51.7)
More than 5 days a week	47 (5.4)
Part-time	373 (42.9)
**Type of service**	
NHS	62 (11.7)
Private	52 (9.8)
Mixed	415 (78.5)

### Dental careers

In total, 41.3% of respondents reported that their career plans were ‘as envisaged', with more than half (58.1%) indicating their career plans had changed over time (online Supplementary Table 1), and 42.2% were planning on changing their careers in future.

In view of the current and future career plans, there were significant differences in demographic factors (sex, ethnicity), country of initial qualification, working experience, primary role setting and job satisfaction (online Supplementary Table 1). A higher proportion of men reported that their career was ‘as envisaged' than women (47.0% versus 37.9%). All ethnic groups, except people of white ethnicity, mostly reported that their careers were not ‘as envisaged'. The proportion of overseas graduates reporting ‘career not as envisaged' (p <0.001) and ‘had changed their career plans' (p = 0.003) was significantly higher than UK graduates. People with a higher duration in their primary job role had a significantly higher proportion of reporting their carer plan was as envisaged (50.8%) and had no plans on changing their careers (47.4%). A significantly higher number of dentists in primary care (44.4%) reported wanting to change their career plans than the rest of the groups in secondary care, university, community dental services and armed forces; a similar trend was seen for those with one job and no further roles in other settings (43.6%) compared with those with additional roles. Significantly higher average job satisfaction scores were evident among respondents whose career was ‘as envisaged' (score 8/10), had an ‘unchanged career plan' (score 8/10) and ‘no plans in changing career' (score 8/10).

Three separate models were used to quantify the associations between predictors and career plan outcomes - career as envisaged, changed career plan and planning on changing career (online Supplementary Table 2). The model fitness was assessed using the likelihood ratio chi-squared test, which showed a significant relationship between the dependent variable and independent variables in all three models (p <0.001). The Pearson and deviance statistics test also indicated that all the models fit the data well (p >0.05). For career as envisaged (Model I), the likelihood ratio test showed that job satisfaction (p <0.001) and primary role setting (p <0.001) contributed significantly to the model. For changed career plan (Model II), job satisfaction (p <0.001), country qualification was obtained (p = 0.026) and primary role setting (p = 0.028) were significant predictors in the model. Job satisfaction (p <0.001) and duration of primary job role (p = 0.005) contributed significantly to Model III (planning on changing career).

In Model I (online Supplementary Table 2), as job satisfaction increased by one unit, the chances of having ‘career not as envisaged' reduced by 0.75 times (95% CI: 0.70-0.81) compared with participants whose careers were as envisaged after adjusting for the effect of other covariates. This means that people who rated higher job satisfaction are more likely to have their career as envisaged than not as envisaged. Male participants were less likely to have their careers not ‘as envisaged' (OR = 0.66; 95% CI: 0.46-0.93) compared to female participants. In other words, the model suggests that males have higher odds of having their careers ‘as envisaged' than females. In addition, participants from primary and secondary care had 0.17 (95% CI: 0.06-0.45) and 0.18 (95% CI: 0.06-0.50) times the odds of having their careers not as envisaged than as envisaged, respectively, compared to armed forces/other settings.

In Model II (online Supplementary Table 2), exploring whether career plans had changed, the odds of an unchanged career plan increased by 17% (95% CI: 1.09-1.25) for each additional unit of job satisfaction score compared with those who changed career plan when controlling for other variables. In other words, people with higher job satisfaction are more likely to report unchanged career plans. UK graduates are more likely to have unchanged career plans (OR = 2.14; 95% CI: 1.11-4.11) compared with participants qualifying abroad. When comparing participants who had no experience working elsewhere with those who had working experience outside and within the UK, the chances of being uncertain whether their career plan had changed increased by 3.41 times (95% CI: 1.14, 10.25) than being sure that their career plan had changed.

In Model III (online Supplementary Table 2), considering changing career plans, participants that scored higher in job satisfaction were 34% (95% CI: 1.24-1.45) more likely to have no intention/plan on changing their careers. When compared with those who had been working for more than 21 years, the odds of all those earlier in their career were over twice as high, with those at 6-10 years 2.82 times (95% CI: 1.30-6.15) more likely to report being uncertain.

### Job satisfaction

There was a significant difference in job satisfaction between different ethnicities, country of initial qualification, primary role settings, work experience, current main role and number of roles (p <0.05) (online Supplementary Table 1). On average, higher job satisfaction was observed in white people, UK graduates, people working outside of primary care settings, specialists, and dentists with extended skills, as well as people with two job roles providing diversity in their career.

Table 2 showed significant associations between job satisfaction and ethnicity, duration of job role, primary settings, current role, number of roles, and career plans. In the adjusted model, dentists' job satisfaction score increased by 1.05 points (95% CI: 0.47-1.63) in people of white ethnicity and 0.71 points (95% CI: 0.54-1.37) in people of Asian ethnicity compared with ‘others'. Participants with more than 20 years of working experience had an increased job satisfaction score by 0.48 points (95% CI: 0.05-0.91) when compared with those with 0-5 years of experience. All other type of role settings showed increased job satisfaction score when compared with working in primary care. Specialists and dentists with extended skills had a higher job satisfaction score by 0.99 points (95% CI: 0.03-1.95) and 0.82 points (95% CI: 0.21-1.42), respectively, when compared with general dental practitioners. Job satisfaction score increased by 0.45 points (95% CI: 0.10-0.81) in people with two job roles as opposed to those with no extra roles. Lastly, job satisfaction score decreased by 1.46 points (95% CI: -1.88 to -1.05) in participants whose career was ‘not as envisaged' but increased by 1.08 points (95% CI: 0.72-1.43) in those not planning to change their careers in the future.

**Table 2:** Multiple linear regression model for job satisfaction

**Adjusted model**	**Coefficient [95% CI]**
**Sex**	
Female	[ref]
Male	-0.22 [-0.55, 0.12]
**Ethnicity**	
Others	[ref]
White	1.05 [0.47, 1.63]***
Multiple ethnicity	0.07 [-1.20, 1.35]
Asian/Asian British	0.71 [0.54, 1.37]*
Black/African/Caribbean/Black British	0.64 [-0.84, 2.13]
**Country obtained qualification**	
Within UK	[ref]
Outside UK	0.23 [-0.34, 0.81]
**Duration of primary job role**	
0-5 years	[ref]
6-10 years	-0.22 [-0.74, 0.30]
11-15 years	-0.10 [-0.61, 0.40]
16-20 years	0.06 [-0.49, 0.60]
21+ years	0.48 [0.05, 0.91]*
**Primary role setting**	
Primary care	[ref]
Secondary care	1.71 [1.04, 2.38]***
University	2.02 [1.19, 2.84]***
Community dental services	1.43 [0.71, 2.15]***
Armed forces/other	1.93 [0.97, 2.90]***
**Experience working in other places**	
Within UK only	[ref]
Outside and within UK	-0.43 [-0.99, 0.14]
Nowhere else	-0.32 [-0.71, 0.07]
**Place of current work**	
Within UK only	[ref]
Outside and within UK	-1.19 [-2.84, 0.45]
Not currently in employment	-1.88 [-3.23, -0.53]^**^
**Current main role**	
General dental practitioner	[ref]
Dentist with special interest/extended skills	0.82 [0.21, 1.42]^**^
Specialist	0.99 [0.03, 1.95]*
Other	2.26 [0.86, 3.65]^**^
**Further roles in other settings**	
No further roles	[ref]
1 role	0.45 [0.10, 0.81]*
≥2 roles	0.29 [-0.45, 1.03]
**Employment**	
Full-time (5 days a week)	[ref]
More than 5 days a week	0.02 [-0.69, 0.72]
Part-time	-0.16 [-0.50, 0.17]
**Type of service**	
NHS	[ref]
Private	0.52 [-0.29, 1.34]
Mixed	0.12 [-0.43, 0.67]
**Career as envisaged**	
Yes	[ref]
No	-1.46 [-1.88, -1.05]***
Unsure	-0.13 [-0.67, 0.40]
**Career plan changed**	
Yes	[ref]
No	-0.10 [-0.51, 0.32]
Unsure	-0.39 [-1.01, 0.22]
**Planning to change career**	
Yes	[ref]
No	1.08 [0.72, 1.43]***
Unsure	0.16 [-0.30, 0.62]
Key: * = p-value <0.05 ** = p-value <0.01 *** = p-value <0.001 CI = confidence interval ref = reference	

## Discussion

### Summary of findings

Overall, our findings suggest that sex, job satisfaction, primary role, country of qualification and duration of working experience impact dentists' career plan trajectories, whether ‘as envisaged', they had ‘changed plan' or were ‘planning on changing'. Women were more likely to report not having had a career plan as envisaged when compared with men, as were dentists with qualifications from outside the UK. Compared with dentists in the latter half of their careers (over 21 years of work experience), junior dentists were more likely to express uncertainties about career changes.

Job satisfaction was associated with having a career as envisaged, not having changed career plan, and not planning to do so. Furthermore, job satisfaction was positively correlated with being of white and Asian ethnicities, longer duration of job role (particularly those with more than 20 years of experience), career progression (specialist and dentists with extended skills) and holding multiple job roles. Conversely, working in primary care was associated with lower job satisfaction.

### Careers

More than half (58.1%) of responding dentists reported that their career plans had changed over time, with just under half (40.2%) planning on changing their careers in the future; however, it is worth noting that career change can be both a positive and negative experience. Furthermore, career choice is not a one-off event, rather a life-course approach to career development. Careers may change throughout people's lives, with different external influences, such as family circumstances and the labour market.^39^^,^^40^^,^^41^ It is important, therefore, to understand how career plans may change and how career decisions are made,^42^ as this can then ideally enable policymakers to facilitate the dental workforce engagement in serving the needs of the population. Retaining a satisfied workforce is important to provide dentistry to the population. It is notable that 20% of those who wanted to change career were interested in leaving the dental workforce. Among this group, more than half (57.5%) were within the first 15 years of their careers.

Family responsibilities and sex were themes raised when participants were asked about influencing factors on their careers. It has been acknowledged that women in dentistry aspire to maintain a family-work balance, alongside having a successful and progressive career across cultures.^19^^,^^43^ However, free-text comments from participants highlighted that some participants found career progression was challenging alongside having a family due to needing to take time out for training, an inability to relocate for training posts with a family, and a need for part-time or flexible working to help manage a work-family balance. This can possibly be explained by longer-term career intentions to achieve specialised or specialist status.^36^

It is important that the profession and policy takes the challenges felt by women seriously, as there has been a transition to a female majority within dentistry, with resultant implications on both the profession and ultimately, patients.^2^ This will ensure we can retain our workforce, particularly as two-thirds of dental students are female,^44^ plus a majority of the workforce.^2^ Facilitating flexible working and training opportunities will allow for improved work-family balance. Flexible training opportunities to improve wellbeing, reduce burnout, and improve access and widen participation to positively impact training opportunities, are now supported nationally.^45^^,^^46^ It is hoped that providing alternative training pathways, including training hubs across the country, will help overcome some of the barriers which exist to accessing further education and training.^47^

### Job satisfaction

It is interesting to note that job satisfaction was significantly higher in individuals who are working outside of primary care settings, hold more than one role and are either a specialist or have extended skills. Research shows that job crafting improves the job role and person fit, with a resultant improvement in job satisfaction.^47^^,^^48^ Providing further education and training for dental professionals is therefore an important aspect of improving workforce satisfaction, with links to improved wellbeing and the resultant improved workforce retention.^48^^,^^49^ Maintaining satisfaction is of particular importance when looking at the primary care workforce as the majority of dentists work in this group. It is felt that significant contract reform is necessary to maintain a viable primary care service.

This is important to consider in today's climate, with the current workforce recruitment and retention crisis in the UK, particularly in rural and coastal areas.^50^^,^^51^ This is highlighted in the recent Nuffield Foundation report,^52^ which emphasises the widespread problems with accessing an NHS dentist and the need for a fundamental reform with a dental recovery plan.

The reasons behind work satisfaction being significantly different for professionals of different ethnicities and country of initial qualification requires further research. However, job satisfaction increased over the professional lifetime, with later-career dentists being significantly more satisfied than early-career dentists. There could be many reasons for this. As dentists progress, they may be developing further skills and become more satisfied with experience, or perhaps those who were previously dissatisfied may have left the profession and are therefore not represented within this sample. Further research should ideally start with obtaining a deeper understanding of what happens in the first phase of dental careers.

Job satisfaction was higher among dentists whose careers were as envisaged. Those who were not planning to change their career in the future were satisfied with their current career choices, yet those who planned to change their career had less job satisfaction, as they are still on their career journey. However, it is important to remember that a career change will not necessarily ensure job satisfaction, as there can be both negative and positive career changes, and this study showed that there was no significant association between job satisfaction and changed career plans. Overall, considering this research was completed during the COVID-19 pandemic, the level of career satisfaction was fairly high, and this is consistent with other dental professionals who reported high job satisfaction with career development opportunities available to them.^53^

### Strengths and limitations

While a large volume of dentists responded, this still represented less than 2% of the profession. Nonetheless, it was broadly representative of registrants. First, there was a relatively even, and comparable, spread across age groups; however, the ratio of male-to-female respondents (367, 42%; 483, 58%) was slightly lower than that for GDC registrants in August 2023 (49%; 51%). Both specialists (10%) and generalists (90%) were represented, in parallel with the GDC register, albeit not necessarily representative at specialty level.^1^ However, there was over-representation of dentists of white ethnicity. Junior colleagues in hospital environments were overrepresented, which can be explained by their possible interest in the subject and HEE (NHS England training and education database) having been one of the distributing organisations. Additionally, it is likely to reflect their interest in the subject matter, with more engaged dentists participating or those having a particularly negative view.^38^ Finally, we did not include foundation dentists in this survey, as they were still at an early stage of their career. Their plans for the following year may have changed as the outcome of national recruitment for the following year may not have been published when they completed the survey, which may have resulted in a change in plans. Further research among this group would be helpful moving forwards.

### Instrument

Second, there are also limitations when asking about career plans as, for example, subject response may be influenced by reader interpretation of the question, participant mood and current employment status. It has been shown that job overload, support and personal development are factors which can influence an individual's perception of the phenomena under study.^54^ Therefore, it will be important to follow this up with in-depth interviews to gain a deeper understanding of professional careers and consider repeating the survey in the future, which would help account for the impact of external factors, such as the COVID-19 pandemic.

Given that the greater majority of GDC-registered dentists are female,^1^ with two-thirds of dental students entering dental school now being female,^44^ issues related to sex need to be taken seriously in light of workforce recruitment and retention, and more research is required to ensure the profession can be supported appropriately to facilitate retention, health and wellbeing, and job satisfaction.

## Conclusion

Careers were not necessarily as envisaged, with over half of the dentists surveyed changing their career plans over their working life. There was greater satisfaction among dentists whose careers were as envisaged and had been able to facilitate career progression. To build on these findings and enhance our understanding of this field, further research should investigate the long-term effects of these factors on dentists' career pathways, the implications for dental education and training, and the development of targeted interventions to support dentists in attaining their career goals and aspirations and ensuring a suitable workforce to meet the needs of our population.

## Supplementary Information

Supplementary Tables 1–2 (PDF 245KB)

## Data Availability

Data supporting this study cannot be made available as participants were not invited to provide consent for the data to be shared publicly.
