# Supplementary material for: Dental careers: findings of a national dental workforce survey
Source: Br Dent J. 2025 Feb 28;238(4):249–56. doi: 10.1038/s41415-024-8234-6 (PMC11870842; doi:10.1038/s41415-024-8234-6)
Supplement: Supplementary file 1 — Supplementary Tables 1–2 (PDF 245KB) [file 41415_2024_8234_MOESM1_ESM.pdf]

Supplementary Table 1 Characteristics of study participants by career plan outcomes and job satisfaction

Characteristics	Career as envisaged				Changed career plan				Planning on changing career				Job satisfaction	
	Yes	No	Unsure	p-value	Yes	No	Unsure	p-value	Yes	No	Unsure	p-value	Median (IQR)	p-value
	n (%)	n (%)	n (%)		n (%)	n (%)	n (%)		n (%)	n (%)	n (%)			
**Total, n**	361 (41.3)	404 (46.2)	109 (12.5)		508 (58.1)	298 (34.1)	68 (7.8)		348 (40.2)	368 (42.5)	150 (17.3)			
**Sex**				0.014				0.016				0.103		0.077
Male	172 (47.0)$_{a}$	147 (40.2)$_{b}$	47 (12.8)$_{a,b}$		202 (55.2)$_{a,b}$	143 (39.1)$_{b}$	21 (5.7)$_{a}$		146 (40.4)$_{a}$	162 (44.9)$_{a}$	53 (14.7)$_{a}$		7 (4)	
Female	184 (37.9)$_{a}$	242 (49.9)$_{b}$	59 (12.2)$_{a,b}$		288 (59.4)$_{a,b}$	151 (31.1)$_{b}$	46 (9.5)$_{a}$		188 (39.0)$_{a}$	200 (41.5)$_{a}$	94 (19.5)$_{a}$		7 (4)	
Not disclosed	5 (21.7)$_{a}$	15 (65.2)$_{a}$	3 (13.0)$_{a}$		18 (78.3)$_{a}$	4 (17.4)$_{a}$	1 (4.3)$_{a}$		14 (60.9)$_{a}$	6 (26.1)$_{a}$	3 (13.0)$_{a}$			
**Ethnicity**				0.025				0.095				0.008		<0.001
White	279 (45.1)$_{a}$	261 (42.2)$_{b}$	78 (12.6)$_{a,b}$		342 (55.3)$_{a}$	229 (37.1)$_{b}$	47 (7.6)$_{a,b}$		240 (39.2)$_{a,b}$	276 (45.1)$_{b}$	96 (15.7)$_{a}$		7 (3)	
Multiple ethnicity	7 (43.8)$_{a}$	7 (43.8)$_{a}$	2 (12.5)$_{a}$		10 (62.5)$_{a}$	5 (31.3)$_{a}$	1 (6.3)$_{a}$		4 (25.0)$_{a}$	10 (62.5)$_{a}$	2 (12.5)$_{a}$		7 (3)	
Asian/Asian British	54 (34.2)$_{a}$	82 (51.9)$_{a}$	22 (13.9)$_{a}$		97 (61.4)$_{a}$	46 (29.1)$_{a}$	15 (9.5)$_{a}$		58 (37.2)$_{a}$	59 (37.8)$_{a}$	39 (25.0)$_{b}$		7 (3)	
Black/African/Caribbean/Black British	2 (18.2)$_{a}$	8 (72.7)$_{a}$	1 (9.1)$_{a}$		9 (81.8)$_{a}$	1 (9.1)$_{a}$	1 (9.1)$_{a}$		5 (45.5)$_{a}$	5 (45.5)$_{a}$	1 (9.1)$_{a}$		6 (3)	
Others	19 (28.8)$_{a}$	41 (62.1)$_{b}$	6 (9.1)$_{a,b}$		48 (72.7)$_{a}$	15 (22.7)$_{a}$	3 (4.5)$_{a}$		38 (57.6)$_{a}$	17 (25.8)$_{b}$	11 (16.7)$_{a,b}$		6 (4)	
**Country obtained qualification**				<0.001				0.003				0.123		<0.001

Within UK	332 (43.2)$_{a}$	337 (43.9)$_{b}$	99 (12.9)$_{a,b}$		433 (56.4)$_{a}$	277 (36.1)$_{b}$	58 (7.6)$_{a,b}$		300 (39.4)$_{a}$	332 (43.6)$_{a}$	129 (17.0)$_{a}$		7 (3)	
Outside UK	29 (27.9)$_{a}$	67 (64.4)$_{b}$	8 (7.7)$_{a,b}$		74 (71.2)$_{a}$	20 (19.2)$_{b}$	10 (9.6)$_{a,b}$		48 (46.6)$_{a}$	34 (33.0)$_{a}$	21 (20.4)$_{a}$		6 (4)	
**Duration of primary job role**				0.004				0.052				0.001		0.067
0–5 years	126 (39.6)$_{a}$	157 (49.4)$_{a}$	35 (11.0)$_{a}$		190 (59.7)$_{a}$	99 (31.1)$_{a}$	29 (9.1)$_{a}$		135 (43.1)$_{a,b}$	112 (35.8)$_{b}$	66 (21.1)$_{a}$		7 (2)	
6–10 years	37 (34.6)$_{a}$	55 (51.4)$_{a}$	15 (14.0)$_{a}$		68 (63.6)$_{a}$	30 (28.0)$_{a}$	9 (8.4)$_{a}$		34 (32.1)$_{a}$	46 (43.4)$_{a}$	26 (24.5)$_{a}$		6 (4)	
11–15 years	39 (33.3)$_{a}$	67 (57.3) $_{a}$	11 (9.4)$_{a}$		76 (65.0)$_{a}$	32 (27.4)$_{a}$	9 (7.7)$_{a}$		41 (35.0)$_{a}$	55 (47.0)$_{a}$	21 (17.9)$_{a}$		7 (4)	
16–20 years	38 (40.0)$_{a}$	43 (45.3)$_{a}$	14 (14.7)$_{a}$		53 (55.8)$_{a}$	34 (35.8)$_{a}$	8 (8.4)$_{a}$		35 (36.8)$_{a}$	43 (45.3)$_{a}$	17 (17.9)$_{a}$		7 (3)	
21+ years	120 (50.8)$_{a}$	82 (34.7)$_{b}$	34 (14.4)$_{a}$		121 (51.3)$_{a}$	102 (43.2)$_{b}$	13 (5.5)$_{a}$		103 (44.0)$_{a}$	111 (47.4)$_{a}$	20 (8.5)$_{b}$		8 (3)	
**Primary role setting**				0.003				0.083				0.008		<0.001
Primary care	231 (42.1)$_{a}$	244 (44.4)$_{a}$	74 (13.5)$_{a}$		318 (57.9)$_{a}$	191 (34.8)$_{a}$	40 (7.3)$_{a}$		241 (44.4)$_{a}$	203 (37.4)$_{b}$	99 (18.2)$_{a,b}$		7 (4)	
Secondary care	76 (51.4)$_{a}$	61 (41.2)$_{a,b}$	11 (7.4)$_{b}$		72 (48.6)$_{a}$	59 (39.9)$_{a}$	17 (11.5)$_{a}$		53 (36.3)$_{a}$	72 (49.3)$_{a}$	21 (14.4)$_{a}$		8 (2)	
University	20 (35.7)$_{a}$	29 (51.8)$_{a}$	7 (12.5)$_{a}$		38 (67.9)$_{a}$	16 (28.6)$_{a}$	2 (3.6)$_{a}$		12 (21.4)$_{a}$	32 (57.1)$_{b}$	12 (21.4)$_{a,b}$		8 (3)	
Community dental services	27 (30.7)$_{a}$	46 (52.3)$_{a}$	15 (17.0)$_{a}$		57 (64.8)$_{a}$	23 (26.1)$_{a}$	8 (9.1)$_{a}$		32 (36.4)$_{a}$	43 (48.9)$_{a}$	13 (14.8)$_{a}$		7 (2)	
Armed forces/other	7 (21.2)$_{a}$	24 (72.7)$_{b}$	2 (6.1)$_{a,b}$		23 (69.7)$_{a}$	9 (27.3)$_{a}$	1 (3.0)$_{a}$		10 (30.3)$_{a}$	18 (54.5)$_{a}$	5 (15.2)$_{a}$		8 (2)	
**Place of current work**				0.659				0.423				0.628		0.405
Within UK only	352 (41.5)$_{a}$	390 (45.9)$_{a}$	107 (12.6)$_{a}$		490 (57.7)$_{a}$	292 (34.4)$_{a}$	67 (7.9)$_{a}$		339 (40.3)$_{a}$	361 (42.9)$_{a}$	142 (16.9)$_{a}$		7 (3)	

Outside and within UK	5 (62.5)$_{a}$	2 (25.0)$_{a}$	1 (12.5)$_{a}$		6 (75.0)$_{a}$	2 (25.0)$_{a}$	0 (0.0)$_{a}$		2 (28.6)$_{a}$	3 (42.9)$_{a}$	2 (28.6)$_{a}$		6 (5.5)	
Not currently in employment	4 (33.3)$_{a}$	7 (58.3)$_{a}$	1 (8.3)$_{a}$		8 (66.7)$_{a}$	4 (33.3)$_{a}$	0 (0.0)$_{a}$		4 (33.3)$_{a}$	4 (33.3)$_{a}$	4 (33.3)$_{a}$		4 (5.5)	
**Experience working in other places**				0.008				0.045				0.481		0.039
Within UK only	74 (41.8)$_{a}$	86 (48.6)$_{a}$	17 (9.6)$_{a}$		105 (59.3)$_{a}$	62 (35.0)$_{a}$	10 (5.6)$_{a}$		69 (39.7)$_{a}$	69 (39.7)$_{a}$	36 (20.6)$_{a}$		8 (3)	
Outside and within UK	43 (30.1)$_{a}$	83 (58.0)$_{b}$	17 (11.9)$_{a,b}$		96 (67.1)$_{a}$	41 (28.7)$_{a}$	6 (4.2)$_{a}$		64 (44.8)$_{a}$	56 (39.2)$_{a}$	23 (16.1)$_{a}$		7 (4)	
No where else	234 (44.5)$_{a}$	221 (42.0)$_{b}$	71 (13.5)$_{a,b}$		290 (55.1)$_{a}$	186 (35.4)$_{a,b}$	50 (9.5)$_{b}$		204 (39.2)$_{a}$	231 (44.3)$_{a}$	86 (16.5)$_{a}$		7 (3)	
**Current main role**				0.364				0.557				0.202		0.039
General dental practitioner	191 (42.8)$_{a}$	197 (44.2)$_{a}$	58 (13.0)$_{a}$		256 (57.4)$_{a}$	156 (35.0)$_{a}$	34 (7.6)$_{a}$		199 (45.1)$_{a}$	156 (35.4)$_{a}$	86 (19.5)$_{a}$		6 (4)	
Dentist with special interest/ extended skills	25 (36.8)$_{a}$	32 (47.1)$_{a}$	11 (16.2)$_{a}$		42 (61.8)$_{a}$	21 (30.9)$_{a}$	5 (7.4)$_{a}$		30 (44.8)$_{a}$	29 (43.3)$_{a}$	8 (11.9)$_{a}$		7 (2)	
Specialist	11 (45.8)$_{a}$	8 (33.3)$_{a}$	5 (20.8)$_{a}$		15 (62.5)$_{a}$	9 (37.5)$_{a}$	0 (0.0)$_{a}$		8 (33.3)$_{a}$	14 (58.3)$_{a}$	2 (8.3)$_{a}$		7.5 (2.5)	
Other	4 (36.4)$_{a}$	7 (63.6)$_{a}$	0 (0.0)$_{a}$		5 (45.5)$_{a}$	5 (45.5)$_{a}$	1 (9.1)$_{a}$		4 (36.4)$_{a}$	4 (36.4)$_{a}$	3 (27.3)$_{a}$		8 (1.5)	
**Further roles in other settings**				0.057				0.100				0.040		<0.001
No further roles	223 (43.1)$_{a}$	239 (46.2)$_{a,b}$	55 (10.6)$_{b}$		281 (54.4)$_{a}$	192 (37.1)$_{b}$	44 (8.5)$_{a,b}$		223 (43.6)$_{a}$	202 (39.5)$_{b}$	86 (16.9)$_{a,b}$		7 (4)	
1 role	104 (38.4)$_{a}$	125 (46.1)$_{a}$	42 (15.5)$_{a}$		173 (63.8)$_{a}$	81 (29.9)$_{a}$	17 (6.3)$_{a}$		87 (32.1)$_{a}$	132 (48.7)$_{b}$	52 (19.2)$_{a,b}$		8 (3)	
≥2 roles	18 (41.9)$_{a}$	15 (34.9)$_{a}$	10 (23.3)$_{a}$		28 (65.1)$_{a}$	13 (30.2)$_{a}$	2 (4.7)$_{a}$		17 (41.5)$_{a}$	17 (41.5)$_{a}$	7 (17.1)$_{a}$		7.5 (3)	
**Employment**				0.665				0.139				0.505		0.185
Full-time (5 days a week)	192 (42.8)$_{a}$	207 (46.1)$_{a}$	50 (11.1)$_{a}$		250 (55.7)$_{a}$	157 (35.0)$_{a}$	42 (9.4)$_{a}$		179 (40.6)$_{a}$	196 (44.4)$_{a}$	66 (15.0)$_{a}$		7 (3)	

More than 5 days a week	17 (36.2)$_a$	22 (46.8)$_a$	8 (17.0)$_a$		24 (51.1)$_a$	20 (42.6)$_a$	3 (6.4)$_a$		18 (38.3)$_a$	19 (40.4)$_a$	10 (21.3)$_a$		7 (3)	
Part-time	149 (40.1)$_a$	173 (46.5)$_a$	50 (13.4)$_a$		232 (62.4)$_a$	118 (31.7)$_a$	22 (5.9)$_a$		150 (40.3)$_a$	152 (40.6)$_a$	71 (19.1)$_a$		7 (3)	
**Type of service**				0.330				0.569				0.166		0.105
NHS	19 (30.6)$_a$	32 (51.6)$_a$	11 (17.7)$_a$		38 (61.3)$_a$	19 (30.6)$_a$	5 (8.1)$_a$		32 (52.5)$_a$	18 (29.5)$_a$	11 (18.0)$_a$		6.5 (5)	
Private	22 (42.3)$_a$	22 (42.3)$_a$	8 (15.4)$_a$		34 (65.4)$_a$	16 (30.8)$_a$	2 (3.8)$_a$		21 (40.4)$_a$	26 (50.0)$_a$	5 (9.6)$_a$		7 (4)	
Mixed	182 (44.0)$_a$	182 (44.0)$_a$	50 (12.0)$_a$		231 (55.8)$_a$	153 (37.0)$_a$	30 (7.2)$_a$		181 (44.1)$_a$	152 (37.1)$_a$	77 (18.8)$_a$		7 (4)	
Job satisfaction$^+$, median (IQR)	8 (3)$_a$	6 (5)$_b$	7 (2)$_a$	<0.001	7 (4)$_a$	8 (3)$_b$	7 (3)$_a$	<0.001	6 (4)$_a$	8 (2)$_b$	7 (4)$_a$	<0.001		

Key:

Chi-squared, Mann-Whitney and Kruskal-Wallis tests were used.

Each subscript letter denotes column proportions whether differ significantly from each other at 0.05 level.

+ Scores out of 10.

IQR: interquartile range.

Supplementary Table 2 Multinomial regression model for career plan outcomes

		Career as envisaged[a] Model I (adjusted) OR [95% CI]	Career plan changed[a] Model II (adjusted) OR [95% CI]	Planning on changing career[a] Model III (adjusted) OR [95% CI]
**No vs Yes[a]**				
	**Sex**			
	Female	[ref]	[ref]	[ref]
	Male	0.66 [0.46, 0.93]*	1.22 [0.88, 1.71]	0.88 [0.61, 1.25]
	Not disclosed	0.96 [0.27, 3.46]	0.67 [0.19, 2.42]	0.562 [0.17, 1.91]
	**Ethnicity**			
	Others	[ref]	[ref]	[ref]
	White	0.63 [0.30, 1.34]	1.34 [0.65, 2.76]	1.37 [0.64, 2.90]
	Multiple ethnicity	0.58 [0.15, 2.30]	1.35 [0.36, 5.12]	7.06 [1.46, 34.21]*
	Asian/Asian British	0.91 [0.40, 2.06]	1.12 [0.50, 2.47]	1.50 [0.65, 3.44]
	Black/African/ Caribbean/Black British	1.65 [0.27, 10.1]	0.35 [0.04, 3.27]	2.34 [0.46, 11.84]
	**Country obtained qualification**			
	Outside UK	[ref]	[ref]	[ref]
	Within UK	0.69 [0.38, 1.28]	2.14 [1.11, 4.11]*	1.18 [0.64, 2.20]
	**Duration of primary job role**			
	21+ years	[ref]	[ref]	[ref]
	0–5 years	1.47 [0.93, 2.32]	0.76 [0.49, 1.16]	0.66 [0.41, 1.04]
	6–10 years	1.37 [0.76, 2.47]	0.76 [0.43, 1.34]	1.42 [0.78, 2.60]
	11–15 years	1.72 [0.99, 2.99]	0.67 [0.39, 1.15]	1.35 [0.77, 2.38]
	16–20 years	1.28 [0.71, 2.33]	1.03 [0.59, 1.79]	1.03 [0.57, 1.86]
	**Primary role setting**			
	Armed forces/other	[ref]	[ref]	[ref]
	Primary care	0.17 [0.06, 0.45]***	1.97 [0.81, 4.76]	0.53 [0.22, 1.30]
	Secondary care	0.18 [0.06, 0.50]***	2.43 [0.96, 6.19]	0.69 [0.27, 1.82]
	University	0.45 [0.15, 1.42]	0.93 [0.32, 2.68]	1.35 [0.44, 4.15]

	Community dental services	0.36 [0.12, 1.06]	1.06 [0.39, 2.90]	0.72 [0.26, 1.98]
	**Experience working in other places**			
	Outside and within UK	[ref]	[ref]	[ref]
	Within UK only	0.90 [0.49, 1.63]	0.82 [0.46, 1.45]	0.83 [0.45, 1.54]
	Nowhere else	0.67 [0.40, 1.13]	1.03 [0.62, 1.70]	1.21 [0.72, 2.05]
	**Job satisfaction**	0.75 [0.70, 0.81]***	1.17 [1.09, 1.25]***	1.34 [1.24, 1.45]***
		**Career as envisaged[a] Model I (adjusted) OR [95% CI]**	**Career plan changed[a] Model II (adjusted) OR [95% CI]**	**Planning on changing career[a] Model III (adjusted) OR [95% CI]**
**Unsure vs Yes[a]**				
	**Sex**			
	Female	[ref]	[ref]	[ref]
	Male	0.77 [0.47, 1.27]	0.60 [0.32, 1.15]	0.64 [0.41, 1.01]
	Not disclosed	1.77 [0.32, 9.88]	1.93 [0.17, 21.51]	0.34 [0.06, 1.87]
	**Ethnicity**			
	Others	[ref]	[ref]	[ref]
	White	0.10 [0.32, 3.13]	7.26 [0.75, 69.97]	1.20 [0.47, 3.02]
	Multiple ethnicity	0.48 [0.04, 5.53]	.	2.52 [0.33, 19.11]
	Asian/Asian British	1.58 [0.46, 5.38]	8.91 [0.88, 89.59]	1.95 [0.74, 5.17]
	Black/African/Caribbean/Black British	2.00 [0.14, 29.49]	4.24 [0.19, 93.20]	0.68 [0.06, 7.37]
	**Country obtained qualification**			
	Outside UK	[ref]	[ref]	[ref]
	Within UK	1.52 [0.56, 4.10]	0.63 [0.24, 1.64]	1.21 [0.57, 2.57]
	**Duration of primary job role**			
	21+ years	[ref]	[ref]	[ref]
	0–5 years	1.02 [0.54, 1.92]	1.14 [0.49, 2.62]	2.13 [1.13, 4.03]*
	6–10 years	1.16 [0.52, 2.59]	1.01 [0.36, 2.82]	2.82 [1.30, 6.15]**
	11–15 years	0.79 [0.34, 1.85]	0.98 [0.36, 2.69]	2.46 [1.14, 5.33]*
	16–20 years	1.26 [0.58, 2.75]	1.23 [0.43, 3.52]	2.22 [1.00, 4.94]*

	**Primary role setting**			
	Armed forces/other	[ref]	[ref]	[ref]
	Primary care	0.86 [0.16, 4.54]	2.11 [0.26, 17.22]	1.22 [0.32, 4.73]
	Secondary care	0.43 [0.07, 2.51]	4.26 [0.50, 36.08]	0.98 [0.23, 4.08]
	University	1.34 [0.21, 8.45]	1.20 [0.10, 14.68]	2.92 [0.61, 13.98]
	Community dental services	1.59 [0.27, 9.28]	2.39 [0.27, 21.40]	1.06 [0.24, 4.71]
	**Experience working in other places**			
	Outside and within UK	[ref]	[ref]	[ref]
	Within UK only	0.54 [0.22, 1.32]	1.63 [0.46, 5.76]	1.42 [0.65, 3.08]
	Nowhere else	0.76 [0.36, 1.58]	3.41 [1.14, 10.25]*	1.25 [0.63, 2.48]
	Job satisfaction	0.95 [0.85, 1.06]	0.96 [0.86, 1.07]	1.04 [0.95, 1.13]

Key:

[a]reference category: Yes

OR = odds ratio

CI = confidence interval

ref = reference

*p-value = <0.05

**p-value = <0.01

***p-value = <0.001
